# Rapid Nanoparticle Synthesis by Magnetic and Microwave Heating

**DOI:** 10.3390/nano6050085

**Published:** 2016-05-05

**Authors:** Viktor Chikan, Emily J. McLaurin

**Affiliations:** Department of Chemistry, Kansas State University, 213 CBC Building, Manhattan, KS 66506-0401, USA

**Keywords:** nanoparticle synthesis, quantum dot synthesis

## Abstract

Traditional hot-injection (HI) syntheses of colloidal nanoparticles (NPs) allows good separation of the nucleation and growth stages of the reaction, a key limitation in obtaining monodisperse NPs, but with limited scalability. Here, two methods are presented for obtaining NPs via rapid heating: magnetic and microwave-assisted. Both of these techniques provide improved engineering control over the separation of nucleation and growth stages of nanomaterial synthesis when the reaction is initiated from room temperature. The advantages of these techniques with preliminary data are presented in this prospective article. It is shown here that microwave assisted heating could possibly provide some selectivity in activating the nanomaterial precursor materials, while magnetic heating can produce very tiny particles in a very short time (even on the millisecond timescale), which is important for scalability. The fast magnetic heating also allows for synthesizing larger particles with improved size distribution, therefore impacting, not only the quantity, but the quality of the nanomaterials.

## 1. Introduction

Colloidal semiconductor nanoparticles (NPs) or quantum dots (QDs) are an important class of materials to address questions in fundamental and practical science [1,2,3,4,5,6,7,8,9,10]. These materials are developed in laboratories where reaction conditions such as mixing (mass transport) and heating (heat transport) can be controlled very precisely to achieve good crystallinity and size distribution. There is a strong impetus to scale up laboratory synthesis to industrial quantities. Traditional colloidal syntheses of QDs, such as the hot-injection (HI) method are not readily scalable [11]. The challenge is that nucleation and growth of the colloidal materials takes place on a time scale comparable to or shorter than the time it takes to mix materials in large reactors or to transfer heat convectively or diffusively.

**Figure 1:**
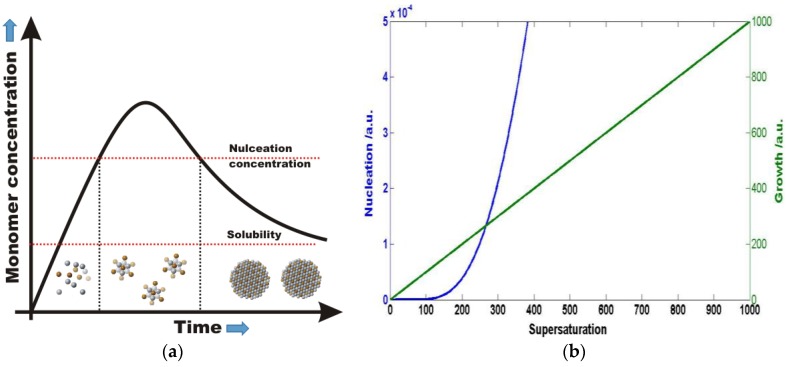
(**a**) LaMer diagram for the nucleation an growth of nanocrystals. (**b**) Dependence of nucleation and growth rate of crystallization on monomer oversaturation S (S = 1 is the solubility of the monomer at any given temperature).

A typical colloidal NP synthesis utilizes traditional crystallization techniques [12]. The goal is to form particles with a uniform size, and therefore uniform physical and chemical properties. The nucleation and growth of the particles are described by LaMer burst nucleation theory [13]. Qualitatively, in solution small nuclei form as a result of significant increase in the monomer concentration, which is followed by a relatively slow growth process. Nucleation and growth can take place simultaneously or separately depending on monomer oversaturation. According to classical nucleation and growth theory from homogenous solution, the rates of nucleation and growth [14] of particles strongly depend on the monomer supersaturation in solution (Figure 1). With a more careful look, one finds that the nucleation process is more strongly dependent on supersaturation than growth. Therefore, at high supersaturation of monomers nucleation may dominate over growth and at lower supersaturation growth may be much faster than nucleation of new small seed crystals. This difference allows one to separate nucleation and growth, assuming that the supersaturation concentration in the colloidal solution can be rapidly controlled.

One of the most common methods to control monomer oversaturation effectively in nanomaterial synthesis, separating the nucleation and growth steps, is the so called hot-injection (HI) method [4]. The HI method separates nucleation and growth by rapid injection of a solution of a monomer precursor followed by its nearly immediate decomposition. In addition, the temperature drop further increases supersaturation, initially favoring nucleation over crystal growth [15]. At a later stage, the monomer supersaturation rapidly drops and crystal growth takes over. At this stage, the monomer supersaturation still remains relatively high, which can achieve efficient size focusing [16]. The HI method works very well at a laboratory scale, but it poses a serious problem during scale up due to limited heat and mass transport. In the first step of the HI method, the precursor has to mix very rapidly to form a homogeneous mixture of precursors. The challenge is that it is difficult to maintain reproducibility when timescale of mixing is similar to the growth and nucleation of the colloidal nanomaterials.

**Figure 2:**
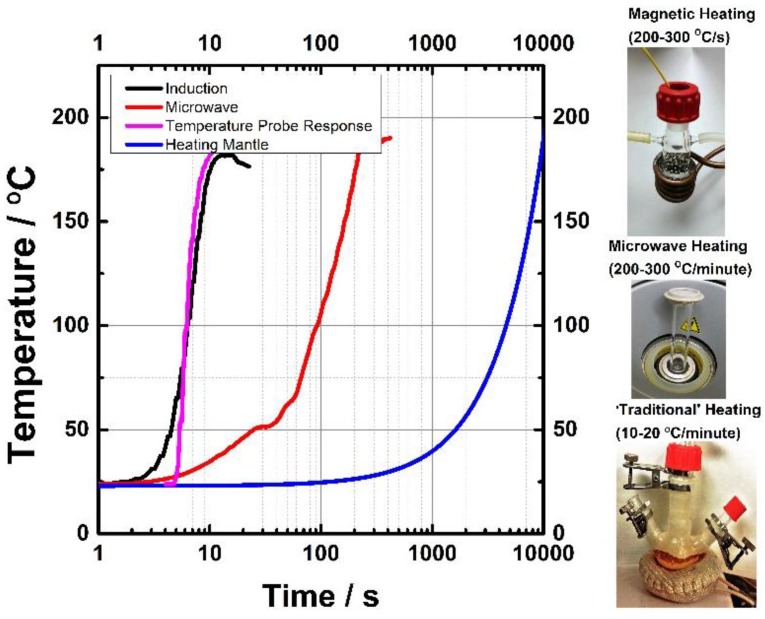
Typical heating rates of magnetic, microwave and traditional heating methods. The heating rates depend on the power absorbed by the reaction mixture or contents.

To minimize mixing problems during the reaction, one can start the synthesis using an already homogeneously mixed solution of precursors. The precursor molecules must decompose very rapidly to create the desirable high monomer supersaturation to separate nucleation and growth [17]. Under these conditions, rapid heating techniques can become a viable alternative to the HI method. The decomposition rate of precursors exponentially depends on the temperature, so the simple solution is to create rapid and uniform temperature rise in the precursor solution. In this paper, proposed solutions are discussed including how the temperature can be increased in the middle of a colloidal solution to reduce the time required for efficient heat transfer. The two specific methods are magnetic and microwave-assisted heating techniques. Due to strong absorption of energy by the contents of the reaction, both of these methods can achieve heating rates significantly higher than traditional laboratory scale (heating mantle) heating methods as shown in Figure 2. Magnetic heating is achieved by either heating a set of steel balls(Bearing-Quality E52100 Alloy Steel, Hardened Ball, 1/8" Diameter) or a magnetic NP solution with a commercially available inductive heater. The actual reactor is shown in Figure 2 also showing the adapter for purging the solution with inert gas such as argon or nitrogen. The reactor with the steel balls is placed into the coil of a 10 kW inductive heater that operates at 366 kHz with a field amplitude of ~5 mTesla. Please note that the actual power delivered to the steel balls will depend on the load and the inductive coils used for the experiment. In our experimental setup with 50 steel balls and with 3 mL of solvent (hexadecylamine or dodecylamine), we can achieve a temperature rise of 300 °C/s in the solution (steel balls + solvent + nanoparticle precursor), producing conditions similar to the HI method. The temperature rise of the reactor is monitored by fiber optic temperature probe with an instrument response time about 500 ms. The temperature rise can be also estimated from the time it takes to get to the boiling point of the solvent. Both methods produce similar temperature rises. Microwave heating can produce about 30 °C/s heating rates, depending on the type of reactor and the type of precursor molecules used for NP synthesis. A rapid temperature rise combined with rapid temperature decrease can lead to the formation of very tiny so-called ultra-small NP. Note, that temperature rise is proportional to the power absorbed in the solution, which is a technical challenge. Here, it is shown that with standard equipment (microwave reactor, magnetically heated reactor, *etc*.) one can produce very rapid temperature jumps needed for nanomaterial synthesis.

## 2. Microwave-Assisted NP Synthesis

Microwave-assisted synthesis is popular in areas ranging from biochemical processes to nanotechnology [18,19,20,21,22,23,24]. Chemical reactions are often faster than traditional convection heating methods, and have higher yields and fewer side products [25,26]. Current microwave reactors provide excellent control over reaction mixing, withstand high temperatures and pressures, and demonstrate exceptional reproducibility from reaction-to-reaction. Speed and efficiency are aided by the direct heating of the reaction mixture (*vs.* convective heating), heating solvents beyond their boiling points at high pressure, and uniform heating profiles [27]. Heating is generally achieved through dipolar polarization and ionic conduction [28]. When the dipoles of irradiated molecules in solution try to align with the oscillating magnetic field, they generate heat. The amount of heat generated is related to the frequency of the field and how fast the molecules align with the field. If alignment is either very fast or very slow, little heating will occur. Ions also move with the oscillating field, colliding and generating heat. These collisions of ions with other species in solution generate much more heat than dipolar polarization. Microwave heating efficiencies can be compared in materials with similar characteristics using the dielectric loss tangent, which is defined as:

tan δ = ε”/ε’

where ε” is the imaginary component of the dielectric, which represents the microwave radiation absorption and conversion to heat, and ε’ is the real component, which signifies the ability of the material to reflect or store an electric field.

For systems, such as NPs, in which the initial parameters, including temperature, heating rate, and precursor reactivity and concentration, define what the nucleation events are, and thus the products, careful attention to the choice of solvent and reactants may yield alternate reaction pathways [21,29]. In addition to heating induced by the precursors/reactants, the reaction products can provide an additional handle for tunability of reaction parameters, namely spatially selective heating [28,30]. Metals and semiconductors can couple with the oscillating magnetic field, inducing a flow of electrons and possible resistive heating. Magnetic NPs can also be affected by other heating mechanisms, including hysteresis, domain wall resonance, and electron-spin resonance. Some studies have looked at these effects, but it is time-consuming to perform the detailed, reproducible studies required for full comprehension [29,31,32,33,34,35,36]. Methods, such as use of a SiC/Au vessel that absorbs microwaves, mimicking heating by convection, can help elucidate these heating processes [37,38].

**Figure 3:**
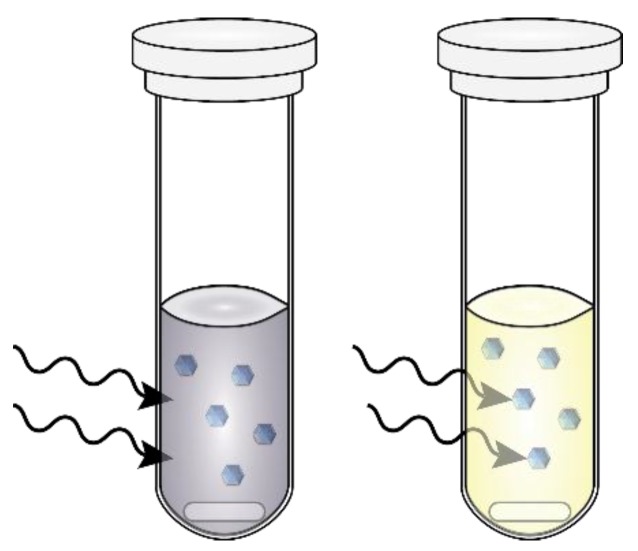
Drawing of two extremes of microwave-assisted reactions. The vial on the left contains a solution with a large tan δ (such as water). Upon microwave irradiation, the microwaves minimally penetrate the reaction volume due to efficient absorption by the solvent. On the right, a reaction with a solvent with a small tan δ (such as an alkane) is shown, and the microwaves can penetrate further into the reaction solution. The reactants in the solution have a larger tan δ, and can interact, increasing temperature.

**Figure 4:**
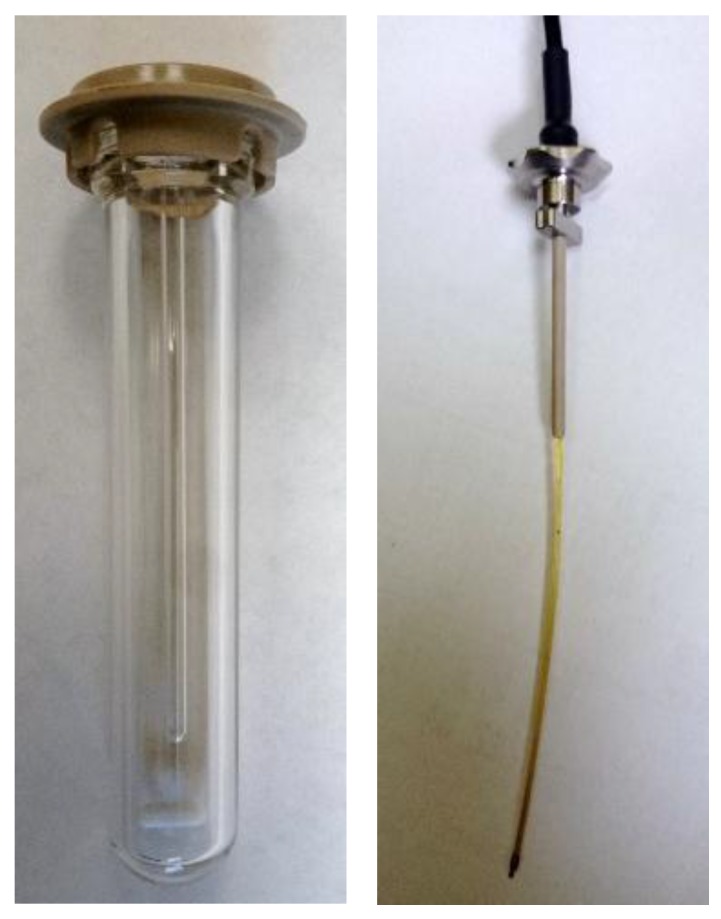
Photographs of a typical 10 mL microwave reaction vessel with a stir-bar and fiber-optic thermometer insert (**left**) and the fiber-optic thermometer (**right**). The glass insert provides values of the internal temperature, but is limited by response time and heat transfer through the insert.

Established methods for the synthesis of semiconductor NPs frequently use high-boiling, hydrophobic solvents (such as octadecene) [39]. These solvents allow for synthesis under inert conditions, reducing the likelihood of formation of defects, but are poor microwave absorbers (have very small loss tangents). Here, we reach a key limitation of microwave-assisted synthesis: rapid heating requires a reaction component with a large tan δ (such as water). This limitation is also an enticing opportunity to explore new reaction space for synthesis of materials with precursors with larger loss tangents than their corresponding reaction media/solvent, a process called selective heating [21,40]. This process and an alternative extreme are shown in Figure 3. On the left, a vial with a solution with a large tan δ efficiently absorbs the irradiated microwaves, heating the reaction. On the right, a solution (solvent) with a small tan δ allows deeper penetration of the microwaves into the reaction volume, and the reactants with larger tan δ can be responsible for increases in temperature [18]. Selective heating has excited synthetic chemists/scientists for decades [25] and, as described here, refers to the generally accepted definition of “microwave effects directly related to heating that result in reactions proceeding via different pathways than their conventional, convective analogs” [21]. In these selective heating reactions, among others, reaction reproducibility is aided by internal temperature measurement, usually with a fiber-optic (FO) probe [41]. This is in contrast to the most popular method for temperature measurement, infrared (IR) detection, which is mostly a read of the vial surface temperature. Figure 4 (left) depicts a typical reaction vial/vessel including a glass insert for a FO probe (right). Internal temperature measurement is a more accurate measure, but many issues can cause deviation from the actual reaction temperature including arcing, field localization around protrusions and interfaces, and other causes of local heating [20,25,42], although these gradients can be minimized with sufficient stirring [41,43].

**Figure 5:**
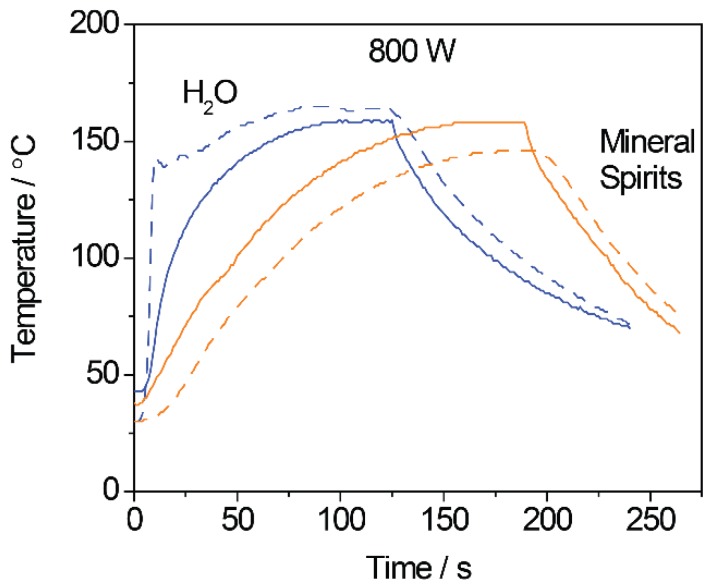
Temperature vs time plots of microwave heating of water (blue) and mineral spirits (orange) to 150 °C at 800 W. The dashed lines indicate the temperatures recorded by the internal fiber-optic thermometer and the solid lines are the temperatures read by the external infrared (IR) sensor.

Discrepancies in temperature readings affect interpretations of NP synthesis especially, due to the heating mechanisms at play [20,44]. In the small tan δ solvents used, like oleylamine or octadecene, temperature measurements from the IR sensor generally overestimate the solution reaction temperature, as the glass vial is heated more than its contents [41]. Alternatively, when the vial contents are strongly absorbing, the IR sensor will tend to underestimate the reaction temperature, measuring the cooler, outside temperature as opposed to that in solution. Molecules and materials with large tan δ have the most apparent discrepancies due to rapid heating of the solution while the pyrex vessel itself remains cooler. This is shown by the temperature-time profile of the heating of water in Figure 5 (blue). The internal temperature sensor reads a value much larger than the external sensor. If the situation is reversed such that a small tan δ molecule/solvent is in the vial, the pyrex vessel will absorb and heat, generating a larger IR temperature reading versus the internal temperature measured by the fiber-optic. This is also seen in Figure 5 (orange). The latter situation is especially relevant to selective heating NP syntheses and key in interpretation of selective heating synthetic results. Thus, reactions showing differences in products obtained by varying microwave parameters must be critically examined for differences in temperature, and used to inform design of microwave-assisted rapid heating pathways.

## 3. Synthesis of Ultra-Small NPs Using Magnetic Heating

**Figure 6:**
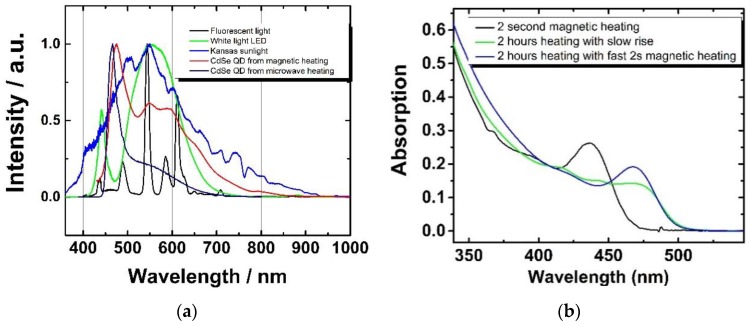
White light emission of ultrasmall quantum dots (QDs) produced in magnetic/microwave heating *vs.* other competing light sources (**a**). Absorption spectra of CdSe QDs synthesized when magnetic heating (**b**) is combined with traditional heating methods. The initial rapid magnetic heating provides a positive impact on the size distribution of the CdSe QDs.

Magneticly assisted nanomaterial synthesis has the potential to produce materials rapidly and at industrial quantities. With the help of magnetic heating techniques, nanomaterials can be produced in a continuous or batch reactor. The key contribution of the magnetic heating is the faster heating rate, which allows processing more material. In a continuous reactor several kilograms of nanomaterials can be synthesized in a few hours’ time with an experimental setup with a small footprint. Using magnetic heating in a batch reactor, ultra-small particles can be produced in a very short amount of time (a few seconds or even on the millisecond timescale). As an example of such a process, single precursor nanomaterial precursors can be decomposed and quenched rapidly to achieve very small sizes of crystals (ultra-small) that are difficult to produce in conventional colloidal synthesis. The Li_4_[Cd_10_Se_4_(SPh)_16¬_] complex is utilized for the production of CdSe QDs at industrial scale [45]. When the Li_4_[Cd_10_Se_4_(SPh)_16¬_] CdSe QD precursor is heated in dodecyl amine with our rapid magnetic heating setup shown in Figure 1, white light emitting CdSe QDs [46] with approximately 10% quantum yield that closely mimic the emission characteristics of natural light (Figure 6) are efficiently produced. Although other groups have produced white light emitting QDs [47,48], the quantum yields of these QDs remains relatively low and their spectral characteristics/spectral stability need further improvement. The magnetic heating is not only able to produce ultrasmall QDs, but is able to influence the initial nucleation to favor narrower size distribution of particles at a later stage of growth. The data show that when the Li_4_[Cd_10_Se_4_(SPh)_16¬_] complex is rapidly heated for two seconds by magnetic induction, the solution contains particles with narrow size distribution. When the solution temperature is maintained at 150 °C with a heating mantle, the CdSe QDs continue to grow to form larger particles. In the absence of rapid preheating from the magnetic heating method described earlier, the solution temperature slowly rises to 150 °C resulting in similar-sized particles, but with broader particle size distribution. The absorption spectra of CdSe QDs produced this way are shown in Figure 6 (right, black and blue). A spectrum of QDs produced using slow heating (light green) shows a broader first absorption feature, indicating a larger size distribution vs the rapidly heated samples.

## 4. Conclusions

In this paper, specific challenges of the scalability of colloidal nanomaterial synthesis in the laboratory are discussed. Two important methodologies are proposed as a potential solution for industrial scale nanomaterial synthesis: Magneticly and microwave-assisted nanomaterial synthesis. Microwave synthesis has the potential to selectively heat either the solvent or the precursor molecules for nanomaterial preparation. In magnetically assisted heating rapid temperature rise allows nanomaterial synthesis to take place in a homogeneously mixed precursor solution. Both of these techniques provide additional engineering control for industrial processing of nanomaterials.

## Abbreviations

The following abbreviations are used in this manuscript:

HI	Hot Injection Method
QD	quantum dot
NP	nanoparticle
FO	fiber optic
IR	infrared
